# The impact of proton pump inhibitors on brain health based on cross-sectional findings from the Rhineland Study

**DOI:** 10.1038/s41598-024-81011-y

**Published:** 2024-12-16

**Authors:** Nersi Alaeddin, Alexandra Koch, Rika Etteldorf, Julia C. Stingl, Monique M.B. Breteler, Folgerdiena M. de Vries

**Affiliations:** 1 Population Health Sciences, German Center for Neurodegenerative Diseases (DZNE), Bonn, Germany; 2 Institute of Clinical Pharmacology, Faculty of Medicine, RWTH Aachen, Aachen, Germany; 3 Institute for Medical Biometry, Informatics and Epidemiology (IMBIE), Faculty of Medicine, University of Bonn, Bonn, Germany

**Keywords:** Public health, Epidemiology

## Abstract

Reports linking proton pump inhibitors (PPIs) with cognition and dementia show conflicting results, with limited evidence on underlying biological mechanisms. However, existing studies did not investigate brain microstructure, which could provide valuable insights into early structural changes indicative of cognitive decline. Analyses were based on cross-sectional baseline data from the Rhineland Study (*n* = 7,465; mean age 55.3 ± 13.7 years, range 30–95 years, 56.5% women). Using multivariate linear regression, we investigated associations between PPI use and cognition and brain macro- and microstructural measures (fractional anisotropy (FA) and mean diffusivity (MD) as measures of white matter integrity). Analyses were stratified by short-term (< 3 years) and long-term (≥ 3 years) PPI use, with additional age stratification (< 65 years; ≥65 years) for cognitive outcomes. PPI users, especially younger individuals, showed poorer global cognition and working memory. Notably, younger long-term users had worse total memory. PPI use was not associated with brain volume or FA, but both short-term and long-term users showed higher MD in cognitive-related brain regions. Our findings indicate that prolonged PPI use, particularly in younger long-term users, is associated with poorer cognitive performance. Moreover, PPI users showed higher MD, indicating potential white matter integrity disruptions. Further research is needed to ascertain causality and underlying mechanisms behind PPI-related cognitive decline.

**Supplementary Information:**

The online version contains supplementary material available at 10.1038/s41598-024-81011-y.

## Introduction

Proton pump inhibitors (PPIs) are commonly prescribed drugs for acid-related gastric disorders that are widely used in Europe and the USA^1,2^. PPIs are available both by prescription and over-the-counter^3,4^. A German study using health insurance data revealed that prescription rates almost doubled between 2005 and 2013 (from 8.2 to 16.2%). This trend showed distinct sex and age disparities, with women using PPIs more than men and older people more than younger people^5^. Evidently, in Germany, PPIs have claimed the 11th place among the most prescribed drugs, with over 5 million prescriptions in 2019^6^. Approximately 40% of these prescriptions are estimated to be inappropriate due to use of PPIs longer than recommended, elevated risk-benefit ratios, and drug-drug interactions^7–9^. This is particularly worrisome, as mounting evidence challenges the presumed safety of long-term PPI use^10^, linking them to various long-term adverse effects, including increased susceptibility to bacterial infections, pneumonia, cardiovascular disease, vitamin deficiencies, bone fractures, chronic kidney disease and even dementia^11–13^.

While there is limited evidence linking PPIs to cognition and dementia via biological mechanisms several hypotheses, although not tested in humans, suggest potential associations with increased risk of dementia: (I) In murine models, PPIs shift the cleavage site of amyloid-β precursor protein (APP), resulting in more β-amyloid_42_ and less β-amyloid_38_^14^; (II) PPIs contribute to a less acidic microglial environment, potentially posing a risk for Alzheimer’s disease by reducing β-amyloid clearance^15^; (III) PPIs inhibit choline acetyltransferase, thereby affecting acetylcholine biosynthesis^16^; (IV) PPI-induced gastric hypoacidity increases susceptibility to prion infections, similar to suggested prion-like mechanisms in Alzheimer’s disease where β-amyloid and tau form plaques and tangles^17–19^. Finally, PPIs reduce the gastric acid needed for vitamin B12 absorption^20^, which has been suggested to contribute to neurodegeneration and cognitive impairment^21^.

Numerous studies have investigated associations between PPIs and dementia, with mixed findings. Some studies suggest an increased risk of dementia with PPI use^22–28^, while others found no discernible risk^29–33^ or, intriguingly, even a reduced risk^34–36^. These conflicting results could be attributed to methodological limitations, including protopathic bias, where the drug is prescribed because of early symptoms of an undiagnosed condition, potentially resulting in erroneous causal inferences between exposure and outcome. Given the long prodromal phase of dementia, this bias holds particular relevance for studies linking PPI use to dementia risk^37,38^. Moreover, the association between PPI use and cognitive decline also remains controversial. While a clinical trial involving sixty young volunteers reported negative effects of PPI use on cognition, other prospective and population-based studies found no such associations^39–42^. In the population-based SHIP study, PPI users scored lower on memory tests, but no association was found between PPI use and brain volume or age^42^. However, this study did not distinguish between short-term and long-term users, potentially biasing effect estimates. Additionally, the study did not examine the effects of PPIs on microstructural brain parameters.

Brain macro- and microstructural measures can help in understanding the potential effects of PPIs on brain health, as they provide detailed insights into the structural integrity and connectivity of the brain. Particularly, microstructural brain imaging through diffusion tensor imaging (DTI) shows promise in detecting early structural changes indicative of cognitive decline. DTI assesses the diffusion rates of water through tissue, revealing hidden variations in tissue integrity that are not visible on conventional MRI scans^43^. The use of imaging techniques that can depict these very early, preclinical changes holds great promise to assess associations between PPI use and brain function. Hence, this study aims to investigate associations between PPI use and cognition, volumetric brain measures, and microstructural DTI measures in the general population.

## Methods

### Study design & setting

The Rhineland Study is a prospective community-based cohort study. Recruitment started in 2016 and is still ongoing. All residents aged ≥ 30 years from two geographically defined areas in Bonn, Germany (Bonn Beuel 40,592 eligible residents; Bonn Hardtberg 36,769 eligible residents- data from July 2022), are eligible. Eligible residents are invited to join the study through both direct invitations and various marketing campaigns to increase awareness and encourage participation. The municipality supports recruitment by providing eligible residents’ contact details. Participation is independent of health status. The only exclusion criterion is insufficient knowledge of the German language to give written informed consent in accordance with the Declaration of Helsinki (all participants provided written informed consent for the study). Participants will be followed for decades, with follow-up examinations every three to four years. All participants undergo in-depth phenotyping, including assessment of cardiovascular health, cognitive testing, MRI scans, neurological function and medication use. The study was approved by the Ethics Committee of the University of Bonn, Medical Faculty.

### Study population

**Fig. 1:**
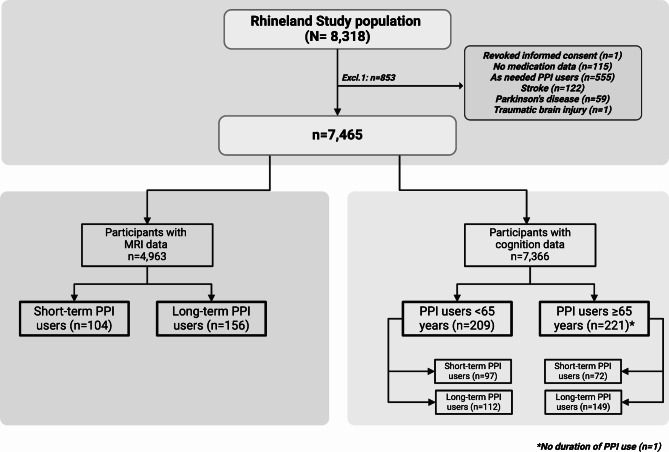
Flowchart of the study population.

For this cross-sectional analysis, we used data from participants who completed baseline examinations between March 2016 and November 2021 (*n* = 8,318). The age distribution of all eligible residents (mean age 56.1 ± 16.0 years) is comparable to the age distribution of those who completed baseline examinations (55.9.8 ± 13.8 years) (p-value 0.574). However, there are slightly more women in the study population compared to the population of eligible residents (53% women vs. 56% women; p-value < 0.001). Of those with completed baseline examinations, we excluded 853 participants because of missing data on PPI exposure (*n* = 115), as needed PPI use (*n* = 555), stroke (*n* = 122), reported Parkinson’s disease (*n* = 59), traumatic brain injury (*n* = 1), and one participant who revoked their informed consent. For the analyses regarding cognition outcomes, we additionally excluded participants without cognitive data (*n* = 99) leaving 7,366 participants, and for the analyses using MRI outcome measures we additionally excluded participants without MRI data (*n* = 2,502) leaving 4,963 participants (Fig. 1).

### Medication data collection and PPI use

Participants were asked to bring the original packages of all medications (including over-the-counter drugs and excluding homeopathic drugs) and prescribed dietary supplements that they were currently taking and had taken as needed in the past year. We registered the name, dosage and Anatomical Therapeutic Chemical (ATC) code of each medication and supplement in an interview.

PPI use was defined as regular use (at regular time intervals e.g. daily, every other day, weekly) of medications starting with ATC code A02BC. Participants who used PPIs on an as-needed basis were excluded from the analyses. Participants who reported not using PPIs were defined as non-users. In addition, PPI users were divided into short-term users (< 3 years) and long-term users (≥ 3 years).

### Cognitive assessment

A battery of cognitive tests was administered according to standardized protocols in German. Details of the cognitive battery are given in Appendix A.

Scores were calculated for different cognitive domains: processing speed, executive function, working memory and episodic verbal memory. Processing speed was assessed using the trail-making test A^44^ (time to task completion) and the eye-tracking prosaccade task (mean saccadic latency, i.e. time needed to initiate a saccade). Executive function was assessed using a categorical word fluency task (total number of animals named), the trail-making test B (time to task completion) and the eye-tracking antisaccade task (percentage of direction errors). Working memory was assessed with a Corsi block-tapping test^45^ and a Digit Span task (maximum forward and backward span). Episodic verbal memory was assessed using the total immediate recall (sum over trials 1–5) and the delayed recall on the Verbal Learning and Memory Test (VLMT)^46,47^. For the cognitive domains, the individual standardized scores within the domains were averaged to calculate the composite score.

An overall memory score was then calculated by averaging the working memory and episodic verbal memory scores, and a global cognitive score was calculated from the average of all cognitive domain scores.

### Magnetic resonance imaging

Magnetic resonance imaging (MRI) was performed on eligible participants using a dedicated 3 Tesla MRI scanner (MAGNETOM Prisma, Siemens Healthineers, Erlangen, Germany) equipped with an 80 mT/m gradient system and a 64-channel phased-array head-neck coil. We assessed the effects of PPI use on total brain volume, cortical grey matter volume, white matter (WM) volume, ventricle volume, hippocampal volume, and cortical thickness. Structural volumes and thicknesses were determined using the standard Freesurfer processing pipeline (http://surfer.nmr.mgh.harvard.edu/) on T1-weighted MR images^48,49^.

We further assessed the effect of PPI use on WM microstructure by using diffusion-weighted MRI (dMRI). DMRI probes brain tissue microstructure by measuring water diffusion properties. We examined fractional anisotropy (FA) and mean diffusivity (MD) obtained by fitting the diffusion tensor model to dMRI scans using the MDT framework. FA is a scalar metric ranging from 0 to 1, with higher values indicating higher anisotropy, i.e. directionality, of water diffusion which is restricted within the complex tissue microstructure in the brain WM. MD is a scalar measure in units of mm^2^/s and reflects the extend of water diffusion, with higher values indicating higher water mobility, i.e. less restriction of water movement within the WM microstructure. Age-related changes in WM microstructure are reflected by decreased FA and increased MD, typically summarized as a decline in WM integrity^50–52^. We examined the average FA and MD values for the entire brain and specifically in the following regions of interest within the brain’s white matter, which have been associated to cognition^53,54^: Body of corpus callosum, left/right cingulum (cingulate gyrus), left/right cingulum (hippocampus), left/right corticospinal tract, fornix (column and body), genu of corpus callosum, left/right posterior thalamic radiation, splenium of corpus callosum, left/right superior longitudinal fasciculus, left/right sagittal stratum (including inferior longitudinal and fronto-occipital fasciculus) and left/right uncinate fasciculus. Further details on MRI acquisition and processing are provided in Appendix B.

### Confounders

The association between PPI use and cognitive and brain measures may be confounded by participant characteristics. Therefore, we simultaneously adjusted for the following characteristics, based on biological plausibility: (I) demographics, including: age (mean-centered), sex, education (based on International Standard Classification of Education (ISCED-11); categorization: low, middle, high), self-reported first-language (native German, non-native), smoking status (never, former, current), body mass index (BMI); (II) comorbidities, including: hypertension (based on current regular use of antihypertensives, and/or mean systolic blood pressure ≥ 140 and/or mean diastolic blood pressure ≥ 90) and diabetes (based on current use of antidiabetic drugs, and/ or Hba1c (no diabetes < 6.5%; diabetes ≥ 6.5%) and/ or fasting glucose (no diabetes < 126 mg/dL; diabetes ≥ 126 mg/dL) measured in morning fasting blood); (III) medication use, including: anticholinergic medication (based on Anticholinergic Burden (ACB) score)^55^, antidepressants, antithrombotic medications, statins, and nonsteroidal anti-inflammatory drugs (NSAIDs); (IV) perceived stress through the Perceived Stress Scale (PSS)^56^.

### Statistical analysis

Descriptive statistics were used to summarize participant characteristics. Group differences were calculated using chi-squared (categorical variables) and ANOVA tests (continuous variables), and separately adjusted for age using logistic regression.

We examined the relation between PPI use and cognitive and brain outcomes using multivariable linear regression. All outcome measures were z-standardized. In the analysis of cognitive domains, we simultaneously adjusted for age, age^2^ (mean-centered to avoid collinearity), sex, education, and the confounders described in Sect. 2.6 as independent variables. In addition, executive function and episodic verbal memory were adjusted for participants’ self-reported first language, as the estimated scores of these two domains are strongly influenced by language proficiency. As age and PPI use yielded a significant interaction term in all cognitive domains, we conducted separate analyses for younger (< 65 years) and older (≥ 65 years) individuals. Analyses were then performed comparing long-term (≥ 3 years) and short-term (< 3 years) PPI use with non-use.

The same approach was used for the brain outcomes, with additional correction for total intracranial volume for volumetric brain outcomes. Here, no age stratification was performed, as there was no significant age interaction term in any of the brain measures. Because we were interested in quantifying the individual relationships between PPI use and cognitive and brain outcomes, rather than testing a joint hypothesis, adjustment for multiple testing would not be warranted^57^. Therefore, we report effect estimates (β) with their respective 95% confidence interval and p-values. All analyses were conducted in RStudio version 4.1.1.

## Results

### Study population

The characteristics of PPI users and non-users are shown in Table 1. Included participants (*n* = 7,465; mean age: 55.3 ± 13.7 years, range 30–95 years; 56.5% women) were younger (*p* < .001) than excluded participants (*n* = 853; mean age: 60.8 ± 14.2 years, range 30–91 years; 55.8% women). Overall, 5.9% (*n* = 444) of the study population were regular PPI users, with more women than men (53.2%) using PPIs. The majority reported long-term (≥ 3 years) PPI use (61.0%) and PPI users were older than non-users (64.7 vs. 54.5 years; *p* < .001). Pantoprazole (*n* = 334) was the most commonly used PPI, followed by omeprazole (*n* = 76), esomeprazole (*n* = 23), lansoprazole (*n* = 9) and rabeprazole (*n* = 4).

**Table 1:** Characteristics of the study population

			Participants with cognition data (n=7,366)				Participants with MRI data (n=4,963)			
	All (n=7,465)	Missing	PPI users (n=431)	PPI non-users (n=6,935)	*p*	Age adjusted *p*	PPI users (n=260)	PPI non-users (n=4,703)	*p*	Age adjusted* p*
Age (years), M (SD)	55.3 (13.7)	0.0%	64.7 (11.6)	54.5 (13.5)	**<0.001**	–	64.3 (11.5)	54.3 (13.4)	**<0.001**	–
Sex (women), N (%)	4,220 (56.5)	0.0%	230 (53.4)	3,932 (56.7)	0.193	0.501	140 (53.8)	2,747 (58.4)	**<0.001**	0.126
Education, N (%)		0.0%			**<0.001**				**<0.001**	
Low	137 (1.8)		21 (4.9)	108 (1.6)		**0.025**	12 (4.6)	66 (1.4)		0.104
Middle	3,306 (44.3)		244 (56.6)	3,010 (43.4)		Ref.	139 (53.3)	1,992 (42.4)		Ref.
High	4,022 (53.9)		166 (38.5)	3,817 (55.0)		**<0.001**	109 (41.9)	2,645 (56.2)		**0.007**
Smoking, N (%)		3.6%			**0.004**				0.088	
Never	3,424 (47.6)		165 (39.6)	3,218 (48.0)		Ref.	107 (42.1)	2,265 (49.1)		Ref.
Former	2,912 (40.5)		194 (46.5)	2,684 (40.1)		0.148	114 (44.9)	1,786 (38.7)		0.438
Current	861 (12.0)		58 (13.9)	796 (11.9)		**0.004**	33 (13.0)	566 (12.3)		0.184
BMI (kg/m^2^), M (SD)	26.0 (4.6)	1.7%	28.1 (4.8)	25.8 (4.6)	**<0.001**	**<0.001**	27.5 (4.3)	25.5 (4.1)	**<0.001**	**<0.001**
Hypertension, N (%)	2,674 (36.0)	0.4%	295 (69.6)	2,308 (33.4)	**<0.001**	**<0.001**	166 (64.6)	1,504 (32.1)	**<0.001**	**<0.001**
Diabetes, N (%)	379 (5.3)	4.5%	56 (13.6)	309 (4.7)	**<0.001**	**<0.001**	28 (11.1)	186 (4.1)	**<0.001**	**0.008**
Medication use, N (%)										
Statins	815 (10.9)	0.2%	129 (30.4)	650 (9.4)	**<0.001**	**<0.001**	72 (27.8)	430 (9.1)	**<0.001**	**<0.001**
Anticholinergics	181 (2.4)	0.5%	33 (8.2)	135 (1.9)	**<0.001**	**<0.001**	22 (8.7)	92 (2.0)	**<0.001**	**<0.001**
Antidepressants	478 (6.4)	0.1%	63 (15.5)	384 (5.5)	**<0.001**	**<0.001**	52 (20.2)	277 (5.9)	**<0.001**	**<0.001**
NSAID	88 (1.2)	0.3%	40 (9.5)	47 (0.7)	**<0.001**	**<0.001**	27 (10.5)	25 (0.5)	**<0.001**	**<0.001**
Antithrombotic	832 (11.2)	0.1%	137 (32.2)	654 (9.4)	**<0.001**	**<0.001**	75 (29.0)	412 (8.8)	**<0.001**	**<0.001**
Cognitive domains (z-standardized)										
Global cognition	0.0 (0.6)	1.3%	− 0.4 (0.6)	0.0 (0.6)	**<0.001**	**<0.001**	− 0.3 (0.6)	0.1 (0.6)	**<0.001**	**0.001**
Total memory	0.0 (0.7)	0.8%	− 0.4 (0.7)	0.0 (0.7)	**<0.001**	**<0.001**	− 0.3 (0.7)	0.1 (0.7)	**<0.001**	**0.008**
Executive function	0.0 (0.8)	0.8%	− 0.4 (0.8)	0.0 (0.7)	**<0.001**	**0.001**	− 0.4 (0.8)	0.0 (0.7)	**<0.001**	**0.021**
Processing speed	0.0 (0.8)	0.4%	− 0.4 (0.9)	0.1 (0.8)	**<0.001**	**0.039**	− 0.4 (0.9)	0.1 (0.8)	**<0.001**	0.050
Working memory	0.0 (0.7)	0.6%	− 0.3 (0.7)	0.0 (0.7)	**<0.001**	**<0.001**	− 0.3 (0.7)	0.0 (0.7)	**<0.001**	**0.009**
Episodic verbal memory	0.0 (0.9)	0.3%	− 0.4 (1.0)	0.1 (0.9)	**<0.001**	**0.003**	− 0.4 (1.0)	0.1 (0.9)	**<0.001**	0.069
Macrostructural brain measures (ml)										
Total brain volume	1106.1 (117.5)	29.1%	1065.5 (112.0)	1109.3 (117.0)	**<0.001**	0.366	1061.4 (109.5)	1107.9 (115.2)	**<0.001**	0.222
Cortical grey matter volume	459.2 (48.0)	29.0%	440.3 (44.9)	460.6 (47.7)	**<0.001**	0.079	438.6 (43.8)	460.1 (47.3)	**<0.001**	**0.042**
White matter volume	456.0 (58.7)	28.9	439.6 (56.5)	457.4 (58.6)	**<0.001**	0.631	437.9 (55.0)	456.8 (57.8)	**<0.001**	0.464
Ventricle volume	28.7 (15.1)	29.6%	35.1 (17.7)	28.3 (14.7)	**<0.001**	0.824	34.6 (17.5)	28.1 (14.4)	**<0.001**	0.994
Hippocampal volume (L hemisphere)	3.8 (0.4)	28.8%	3.7 (0.4)	3.9 (0.4)	**<0.001**	0.747	3.7 (0.4)	3.9 (0.4)	**<0.001**	0.974
Hippocampal volume (R hemisphere)	3.9 (0.5)	28.8%	3.8 (0.5)	3.9 (0.4)	**<0.001**	0.843	3.8 (0.5)	4.0 (0.5)	**<0.001**	0.832
Cortical thickness (L hemisphere)	2.4 (0.1)	29.0%	2.4 (0.1)	2.4 (0.1)	**<0.001**	0.751	2.4 (0.1)	2.4 (0.1)	**<0.001**	0.750
Cortical thickness (R hemisphere)	2.4 (0.1)	29.0%	2.4 (0.1)	2.4 (0.1)	**<0.001**	0.977	2.4 (0.1)	2.4 (0.1)	**<0.001**	0.890
Microstructural brain measures										
Global FA	0.6 (0.0)	32.6%	0.6 (0.0)	0.6 (0.0)	**<0.001**	**0.023**	0.6 (0.0)	0.6 (0.0)	**<0.001**	**0.012**
Global MD (10^-4 mm^2/s)	0.0 (0.0)	32.6%	0.0 (0.0)	0.0 (0.0)	**<0.001**	0.090	0.0 (0.0)	0.0 (0.0)	**<0.001**	0.075

*BMI* Body Mass Index, *FA* fractional anisotropy, *M* mean, *MD* mean diffusivity, *L* left, *N/n* number of participants, *NSAID* non-steroidal anti-inflammatory drugs, *R* right, *Ref* reference, *SD* standard deviation.

Data presented as mean ± standard deviation for quantitative variables/ percentages for categorical variables. *p*-values in bold represent significant values. Differences between PPI users and non-users were assessed with logistic regression adjusted for age.

### Cognition

Across all cognitive domains, we observed a consistent but non-significant pattern of poorer cognitive performance in users of PPIs compared to non-users. Stratified analyses showed that PPI use was significantly associated with worse global cognition (β= -0.07, 95%CI -0.13; -0.01, *p* = .026), and total memory (β=-0.09, 95%CI -0.17; -0.01, *p* = .024), and working memory performance (β= -0.13, 95%CI -0.22; -0.03, *p* = .007), in younger individuals (aged < 65 years) (Table 2). The effect sizes were comparable to an average age-related decline in global cognition, total memory and working memory, of 1.5, 2 and 7.5 years, respectively.

**Table 2:** Linear regression β coefficients (95%CI) for PPI use on z-standardized cognitive domain scores (stratified by age groups and duration of use)

	All (n=7,366; PPI users: n=431)			<65 years (n=5,480; PPI users: n=209)			≥65 years (n=1,886; PPI users: n=222)		
	β	*95% CI*	*p*	β	*95% CI*	*p*	β	*95% CI*	*p*
All PPI users									
Global cognition	−0.04	−0.08–0.01	0.125	**−0.07**	**−0.13–−0.01**	**0.026**	−0.00	−0.07–0.06	0.905
Total memory	−0.04	−0.10–0.02	0.178	**−0.09**	**−0.17–−0.01**	**0.024**	0.01	−0.08–0.09	0.876
Executive function*	−0.03	−0.09–0.04	0.382	−0.07	−0.15–0.02	0.143	0.01	−0.09–0.11	0.837
Processing speed	−0.02	−0.08–0.05	0.584	−0.01	−0.10–0.08	0.817	−0.03	−0.14–0.07	0.537
Working memory	−0.05	−0.12–0.02	0.132	**−0.13**	**−0.22–−0.03**	**0.007**	0.03	−0.07–0.12	0.585
Episodic verbal memory*	−0.02	−0.11–0.05	0.553	−0.05	−0.16–0.06	0.375	−0.01	−0.14–0.12	0.869

	All Short−term users (n=169)			Short−term users <65 years (n=97)			Short−term users ≥65 years (n=72)		
	β	*95% CI*	*p*	β	*95% CI*	*p*	β	*95% CI*	*p*
Short-term users (<3 years)									
Global cognition	−0.02	−0.09–0.05	0.608	−0.07	−0.16–0.02	0.124	0.05	−0.07–0.16	0.424
Total memory	−0.01	−0.10–0.08	0.837	−0.07	−0.18–0.05	0.262	0.06	−0.08–0.21	0.389
Executive function*	0.01	−0.09–0.11	0.777	−0.07	−0.20–0.06	0.288	0.12	−0.05–0.29	0.173
Processing speed	−0.04	−0.15–0.06	0.417	−0.05	−0.18–0.08	0.427	−0.04	−0.22–0.14	0.692
Working memory	−0.04	−0.14–0.06	0.410	−0.13	−0.27–0.00	0.053	0.07	−0.08–0.22	0.363
Episodic verbal memory*	0.03	−0.09–0.16	0.617	0.01	−0.15–0.17	0.907	0.06	−0.16–0.27	0.589

	All Long−term users (n=261)			Long−term users <65 years (n=112)			Long−term users ≥65 years (n=149)		
	β	*95% CI*	*p*	β	*95% CI*	*p*	β	*95% CI*	*p*
Long-term users (≥3 years)									
Global cognition	−0.05	−0.10–0.01	0.110	−0.07	−0.15–0.01	0.091	−0.03	−0.11–0.05	0.511
Total memory	−0.06	−0.13–0.01	0.166	**−0.11**	**−0.22–−0.01**	**0.036**	−0.02	−0.12–0.08	0.730
Executive function*	−0.06	−0.14–0.02	0.143	−0.06	−0.18–0.05	0.287	−0.04	−0.16–0.08	0.499
Processing speed	−0.00	−0.08–0.08	0.950	0.02	−0.09–0.14	0.696	−0.03	−0.16–0.10	0.638
Working memory	−0.06	−0.14–0.02	0.162	−0.12	−0.24–−0.00	0.050	−0.00	−0.11–0.11	0.999
Episodic verbal memory*	−0.06	−0.16–0.05	0.280	−0.10	−0.24–0.05	0.186	−0.04	−0.19–0.12	0.642

Models adjusted for age, age^2^, sex, education, smoking, German mother tongue (*only for executive function and episodic verbal memory), BMI, hypertension, diabetes, antithrombotic medication, antidepressants, statins, anticholinergic medication, NSAIDs, perceived stress.

*BMI* body mass index, *CI* confidence interval, *n* number of participants, *NSAID* non-steroidal anti-inflammatory drugs, *PPI* proton pump inhibitors.

Values in bold represent significant values*.*

In both younger (< 65 years) and older (≥ 65 years) individuals, no significant differences in cognitive performance were observed between short-term PPI users and non-users. However, among younger long-term PPI users, we observed significant negative effects of PPI use, resulting in worse total memory (β= -0.11, 95%CI -0.22; -0.01, *p* = .036) compared to non-users.

### Macro- and microstructural brain measures

We identified no significant differences in any of the assessed brain macrostructural measures (Table 3), nor with global fractional anisotropy and the investigated region-specific FA parameters (data not shown) between PPI users and non-users.

**Table 3:** Linear regression β coefficients (95%CI) for PPI use on z-standardized macrostructural brain measures (stratified by age groups and duration of use)

	All (n=4,963; 260 PPI users)			Short-term PPI users (n=104)			Long-term PPI users (n=156)		
	β	*95% CI*	*p*	β	*95% CI*	*p*	β	*95% CI*	*p*
Total brain volume	0.02	−0.03–0.06	0.435	−0.03	−0.09–0.04	0.469	0.05	−0.01–0.10	0.108
Cortical grey matter volume	−0.01	−0.06–0.05	0.834	−0.05	−0.14–0.04	0.246	0.02	−0.05–0.10	0.516
White matter volume	0.02	−0.04–0.08	0.554	−0.02	−0.11–0.08	0.707	0.04	−0.04–0.12	0.280
Ventricle volume	0.00	−0.10–0.11	0.962	0.07	−0.09–0.23	0.373	−0.04	−0.17–0.09	0.510
Hippocampal volume (left hemisphere)	0.00	−0.10–0.10	0.997	0.04	−0.12–0.19	0.641	−0.02	−0.15–0.10	0.714
Hippocampal volume (right hemisphere)	0.00	−0.10–0.10	0.951	−0.01	−0.16–0.14	0.905	0.01	−0.11–0.13	0.860
Cortical thickness (left hemisphere)	0.03	−0.08–0.15	0.584	−0.02	−0.20–0.16	0.834	0.07	−0.08–0.21	0.374
Cortical thickness (right hemisphere)	0.04	−0.08–0.16	0.524	−0.02	−0.21–0.16	0.814	0.08	−0.07–0.23	0.303

Models adjusted for age, age^2^, sex, education, smoking, BMI, hypertension, diabetes, antithrombotic medication, antidepressants, statins, anticholinergic medication, NSAIDs, perceived stress, estimated intracranial volume.

*BMI* body mass index, *CI* confidence interval, *n* number of participants, *NSAID* non-steroidal anti-inflammatory drugs, *PPI* proton pump inhibitors.

Values in bold represent significant values*.*

We observed higher global MD (β = 0.12, 95%CI 0.01; 0.23, *p* = .039) and elevated MD in the body of the corpus callosum (β = 0.20, 95%CI 0.08; 0.33, *p* = .001) in PPI users compared to non-users.

In subgroup analyses, short-term users showed higher MD in the body of the corpus callosum (β = 0.22, 95%CI 0.03; 0.41, *p* = .022) the left cingulum (hippocampus) (β = 0.23, 95%CI 0.03; 0.43, *p* = .025) and the left uncinate fasciculus (β = 0.24, 95%CI 0.03; 0.44, *p* = .024) compared to non-users (Table 4).

**Table 4:** Linear regression β coefficients (95%CI) for PPI use on z-standardized microstructural brain measures (stratified by age groups and duration of use)

	All (n=4963; 250 PPI users)			Short-term PPI users (n=104)			Long-term PPI users (n=156)		
	β	*95% CI*	*p*	β	*95% CI*	*p*	β	*95% CI*	*p*
Global FA	−0.07	−0.20–0.05	0.261	−0.15	−0.34–0.04	0.118	−0.02	−0.17–0.14	0.813
Global MD	**0.12**	**0.01–0.23**	**0.039**	0.15	−0.02–0.32	0.087	0.10	−0.04–0.24	0.174
Body of corpus callosum (MD)	**0.20**	**0.08–0.33**	**0.001**	**0.22**	**0.03–0.41**	**0.022**	**0.19**	**0.03–0.34**	**0.018**
Left cingulum (cingulate gyrus)	0.11	−0.01–0.23	0.081	0.14	−0.04–0.33	0.134	0.09	−0.07–0.24	0.270
Right cingulum (cingulate gyrus) (MD)	0.09	−0.03–0.22	0.151	0.10	−0.09–0.29	0.308	0.09	−0.07–0.25	0.280
Left cingulum (hippocampus) (MD)	0.05	−0.08–0.18	0.456	**0.23**	**0.03–0.43**	**0.025**	−0.07	−0.23–0.10	0.418
Right cingulum (hippocampus) (MD)	−0.06	−0.20–0.08	0.423	0.15	−0.07–0.36	0.178	−0.19	−0.36–−0.02	0.333
Left corticospinal tract (MD)	0.05	−0.08–0.19	0.453	−0.11	−0.32–0.10	0.303	0.16	−0.01–0.33	0.070
Right corticospinal tract (MD)	0.12	−0.02–0.26	0.099	0.01	−0.20–0.22	0.906	**0.18**	**0.01–0.36**	**0.037**
Fornix (column and body) (MD)	0.00	−0.13–0.13	0.990	0.01	−0.19–0.21	0.938	−0.00	−0.17–0.16	0.964
Genu of corpus callosum (MD)	0.03	−0.10–0.16	0.650	0.04	−0.16–0.24	0.683	0.02	−0.14–0.19	0.785
Left posterior thalamic radiation (MD)	0.00	−0.12–0.12	0.988	0.06	−0.12–0.25	0.494	−0.04	−0.19–0.11	0.598
Right posterior thalamic radiation (MD)	0.03	−0.10–0.15	0.665	0.11	−0.08–0.30	0.266	−0.03	−0.18–0.13	0.751
Splenium of corpus callosum (MD)	0.14	0.01–0.27	0.440	0.08	−0.12–0.28	0.417	0.17	0.01–0.34	0.390
Left superior longitudinal fasciculus (MD)	0.09	−0.02–0.21	0.113	0.11	−0.07–0.29	0.217	0.08	−0.06–0.23	0.268
Right superior longitudinal fasciculus (MD)	0.12	−0.00–0.24	0.051	0.14	−0.04–0.32	0.128	0.10	−0.05–0.25	0.175
Left sagittal stratum (MD)	0.00	−0.11–0.12	0.935	0.00	−0.18–0.18	0.996	0.01	−0.14–0.16	0.918
Right sagittal stratum (MD)	0.06	−0.06–0.19	0.342	0.07	−0.12–0.27	0.443	0.05	−0.11–0.21	0.521
Left uncinate fasciculus (MD)	0.07	−0.06–0.21	0.287	**0.24**	**0.03–0.44**	**0.024**	−0.03	−0.20–0.13	0.687
Right uncinate fasciculus (MD)	0.11	−0.03–0.24	0.122	0.12	−0.08–0.33	0.247	0.10	−0.07–0.26	0.264

Models adjusted for age, age^2^, sex, education, smoking, BMI, hypertension, diabetes, antithrombotic medication, antidepressants, statins, anticholinergic medication, NSAIDs, perceived stress.

*BMI* body mass index, *CI* confidence interval, *FA* fractional anisotropy, *MD* mean diffusivity, *n* number of participants, *NSAID* non−steroidal anti−inflammatory drugs, *PPI* proton pump inhibitors;

Values in bold represent significant values*.*

In long-term users, MD was higher in the body of the corpus callosum (β = 0.19, 95%CI 0.03; 0.34, *p* = .018) and the right corticospinal tract (β = 0.18, 95%CI 0.01; 0.36, *p* = .037) compared to non-users.

## Discussion

In the context of the population-based Rhineland Study, we investigated the impact of PPIs on cognitive function, and macro- and microstructural brain measures. We observed that younger PPI users, particularly those with a longer duration of use, had poorer cognitive performance compared to non-users. While volumetric brain measures showed no discernible differences between PPI users and non-users, we observed a higher mean diffusivity among PPI users in some brain regions, especially the corpus callosum.

Younger PPI users exhibited poorer performance across various cognitive domains, namely global cognition, total memory and working memory, when compared to non-users. These findings align with a small clinical trial conducted by Akter et al. on 60 young participants, reporting impaired cognitive performance in visual memory, attention, executive function and working/ planning function^39^. Further, Ahn and colleagues reported lower VLMT scores in PPI users^42^. In contrast, we did not find a significant association between PPI use and episodic verbal memory. Instead, the most pronounced effect was identified in working memory, which likely also drives the observed association between PPI use and the total composite memory score.

Moreover, we observed poorer cognitive performance in long-term PPI users (< 65 years). This observation aligns with stratified analyses conducted in two age groups of a Danish middle-aged cohort, where a stronger negative effect was seen in participants < 57 years, even though statistical significance was not reached, likely due to the limited number of PPI users in these subsets^41^. The absence of noticeable effects in older participants may be due to competing causes like comorbidities, which can have a more substantial impact on cognitive performance. Another contributing factor could be the increased sensitivity of younger individuals to medications, potentially linked to age-related changes in pharmacokinetics and pharmacodynamics of medications^58^.While there is a potential age-related influence of PPIs on cognitive function, causality remains uncertain.

Several hypotheses link cognitive decline and dementia to biological mechanisms^14–16^. Yet, the relevance of these hypotheses to human health remains uncertain due to lack in human testing. A more accepted theory connects cognitive decline with vitamin B12 deficiency^21^, supported by a case-control study showing an association between gastric acid inhibitors and reduced vitamin B12 absorption^20^. Given PPIs’ impact on vitamin B12 absorption, it is plausible that they can potentially contribute to cognitive decline.

We did not find any significant associations between PPI use and brain volumetric measures, consistent with prior research^42^. However, we observed an association of PPI use with elevated mean diffusivity in certain brain regions linked to cognitive function, suggesting potential adverse effects on brain integrity. MD is a valuable indicator of microstructural changes in brain tissue that can depict early, preclinical changes in brain function. It reflects alterations in water diffusion that can signal axonal and myelin damage not distinguishable using conventional MRI^59^.

Short-term users had higher MD in specific brain regions, such as in the body of the corpus callosum, and the left cingulum (hippocampus), compared to non-users. Long-term users similarly displayed higher MD, particularly in the body of the corpus callosum and the right corticospinal tract. These findings align with our cognitive performance observations in PPI users, given that the aforementioned regions are known to be associated with cognitive function^53^. Additionally, previous research has indicated that increased MD reflects decreased WM microstructure, a phenomenon often observed in the ageing brain or in brains affected by diseases^50–52^. Although our findings also hint at a potential link between PPI use and cognitive impairment^60–62^, it is important to note that our results do not conclusively identify WM as the sole critical factor, necessitating further investigations.

Changes in PPI exposure during the prodromal phase of dementia could falsely show a negative effect of PPI use on cognition and dementia risk. This phase often involves prescription of medications to manage symptoms like depression, anxiety, sleep disturbances and cognitive impairment^63^. Some of these prescribed medications themselves can induce dyspeptic symptoms, for which PPIs are commonly recommended. Additionally, depression and anxiety may themselves cause dyspeptic symptoms^64^, which may lead to PPI use. To address and mitigate these complexities, we implemented several approaches. First, in addition to controlling for a variety of confounding factors, we conducted subgroup analyses within two age groups (younger (< 65 years) and older individuals (≥ 65 years) for the cognitive outcomes. This stratification allowed us to explore potential age-related variations in the effects of PPI use. Furthermore, we differentiated between short-term and long-term PPI use, uncovering duration-dependent impacts that added depth to our analyses.

The strength of our study also lies in its ability to assess the effects of PPI use on both macrostructural and, notably, microstructural measures of the brain, representing a novel contribution to the field. Given the multifactorial potential influences on cognitive and brain function, our detailed phenotyping of our participants enabled us to control for numerous confounding factors. Furthermore, we collected medication data through interviews. This method captured information on both prescription-based and over-the-counter PPIs, enhancing the comprehensiveness of our study. To ensure the reliability of our data, we have previously validated self-reported medication data collected in the Rhineland Study and found a good accuracy, independent of age and sex^65^. Although prescription-based medication exposure is typically the standard in pharmacoepidemiological studies, both self-reported and prescription-based exposures have their limitations. Ideally, combining these two sources would be best, but this is unfortunately rarely possible.

We must also acknowledge certain limitations in our study. Its cross-sectional nature restricted our ability to explore temporal associations, leaving us unable to confirm whether PPI use preceded the observed changes in cognitive performance and brain microstructural measures. Therefore, we cannot definitively rule out the possibility of reversed causality. Second, our population might represent a “healthier” population, potentially limiting the generalizability of our findings. However, our population does not seem healthier than the general German population, as indicated by comparable prevalence rates of polypharmacy and hypertension^66,67^. Third, we did not adjust for multiple testing, as our primary aim was effect estimation, rather than hypothesis testing. Following Rubin’s argument^57^, corrections for multiple testing are crucial in disjunction testing, where the goal is to reject a joint null hypothesis based on at least one significant result. However, presenting multiple associations without such adjustments could increase the likelihood of finding spurious associations due to chance. Therefore, statistically significant associations should be interpreted with caution. A more reliable approach is to consider the 95% CIs, which provide a range within which the true effect likely lies, offering a more robust estimate than focusing solely on p-values. Lastly, as in all pharmacoepidemiological studies, the potential for residual confounding by indication, particularly among long-term users, necessitates consideration. Despite our extensive adjustments, the influence of unmeasured variables on our findings cannot be entirely ruled out.

To conclude, our study reveals negative effects of PPI use on cognition, especially in younger long-term users. We did not find significant associations between PPI use and brain volume, but observed higher mean diffusivity in PPI users in specific brain regions, indicating potential white matter integrity impacts. Highlighting the imperative for deprescribing PPIs, especially in younger individuals, future studies necessitate larger sample sizes and longitudinal data for definitive conclusions. Meanwhile, exercising caution to PPI use is advisable to avoid potential risks of cognitive decline and changes in brain structure.

## Electronic supplementary material

Below is the link to the electronic supplementary material.

Supplementary Material 1

## Data Availability

The datasets for this manuscript are not publicly available because of data protection regulations. Access to data can, however, be provided to scientists in accordance with the Rhineland Study’s Data Use and Access Policy. Requests to access the datasets should be directed to Prof. Dr. Dr. Monique M.B. Breteler, RS-DUAC@dzne.de.
