# Supplementary material for: The impact of proton pump inhibitors on brain health based on cross-sectional findings from the Rhineland Study
Source: Sci Rep. 2024 Dec 16;14:30486. doi: 10.1038/s41598-024-81011-y (PMC11649697; doi:10.1038/s41598-024-81011-y)
Supplement: Supplementary file 1 — Supplementary Material 1 [file 41598_2024_81011_MOESM1_ESM.docx]

**APPENDIX A: Cognitive test battery**

*Verbal Learning and Memory Test*

We used the German version of the 15-word Verbal Learning and Memory Test (VLMT), which is analogous to the Rey Auditory Verbal Learning Test [1,2]. Here, 15 semantically unrelated nouns are learned and recalled over multiple trials, and declarative episodic verbal memory (short- and long-term memory) and learning performance are measured. The test begins with five trials of auditory learning and recall, followed by recall of an interference list, another immediate recall and delayed recall after 20-30 minutes. The outcome measures were the number of words recalled correctly in the immediate recall (sum of recalls one to five) and the number of words recalled correctly after the time delay.

*Digit Span task*

The Digit Span task is a verbal working memory task in which participants were asked to recall sequences of digits of increasing length in forward (sequence length 3-9) and backward order (sequence length 2-9). After two errors within a sequence in the forward and backward tests, the task ends. The maximum sequence length was used as the outcome.

*Corsi block-tapping test*

The Corsi block-tapping test is a verbal working memory test [3]. Participants are asked to recall visuospatial sequences of blocks that changed colour by tapping the blocks in the correct order on a tablet. In the forward task (sequence length 2-9), participants tap the blocks in forward order, and in the backward task (sequence length 2-9), participants tap the blocks in backward order. After two errors within a sequence, the task ended and the length of the last sequence successfully completed was used as the result.

*Trail-making test*

The trail-making test (TMT) assesses processing speed and executive function and has been adapted from the Psychology Experiment Building Language (PEBL) test battery [4] to a touch screen. In version A of the TMT, numbers from 1 to 24 are randomly scattered on the screen and participants need to connect them in ascending order (1-2-3- etc) as quickly as possible. In version B, 12 numbers (1 to 12) and 12 letters (A to L) are scattered randomly on the screen and have to be connected in ascending order and in alternation (1-A-2-B etc) as quickly as possible. The main outcome is the time taken to complete the task in both versions. If the participant takes more than 301 seconds, the test is automatically stopped.

*Word fluency task*

The word fluency task assesses semantic memory and executive function by asking participants to name as many animals as possible in one minute. The score is based on the number of animals named correctly.

*Prosaccade and antisaccade tasks*

The prosaccade and antisaccade tasks are part of the eye-tracking examination [5] and are used as a cognitive measure of attention, processing speed and executive function. In both tasks, a 1° diameter white circle is presented on a black background. In the prosaccade task, a stimulus appeared at the central position for a randomly determined duration. The stimulus moved to a horizontal side position where it remained for 1,000 ms before moving back to the central position (random order of 15 left-sided and 15 right-sided trials). Participants were instructed to follow the stimulus as closely as possible. Subsequently, the antisaccade task was performed in the same way, except that participants were instructed to look at the stimulus when it was in the central position and immediately look at the opposite (mirror) position when the stimulus moved to the side. Prosaccade latency (time needed to initiate a saccade) and antisaccade error rate (percentage of trials, in which the participant makes a direction error) were used as outcome measures.

**APPENDIX B: MRI acquisition and processing**

The 3T MRI scanners (MAGNETOM Prisma, Siemens Healthineers, Erlangen, Germany) are equipped with a 64-channel phased-array head/neck coil. T1-weighted images were acquired with an isotropic spatial resolution of 0.8 mm using a multi-echo MPRAGE sequence (acquisition time = 6.5 min, repetition time = 2560 ms, inversion time = 1100 ms, flip angle 7°, field of view = 256 x 256 mm, 224 sagittal slices). Structural volumes and thicknesses were determined using the standard FreeSurfer processing pipeline (<http://surfer.nmr.mgh.harvard.edu/>) [6,7]  on T1-weighted MR images. We used the estimated total intracranial volume (eTIV) generated by FreeSurfer as a proxy for head size [8].

Simultaneous-multi-slice diffusion weighted MRI (dMRI) was performed using a spin-echo echo-planar imaging (SE-EPI) sequence applying threefold slice-acceleration [9–11]. A compressed sensing [12] diffusion spectrum imaging [13] (CS-DSI) protocol [14] was used to acquire dMRI scans with an isotropic spatial resolution of 1.5 mm (acquisition time = 12.1 min, repetition time = 5500 ms, echo time = 105ms, field of view =210x210mm, 96 slices, diffusion weighting =6800 s/mm2, gradient pulse separation =49.5ms, gradient pulse duration =19.7ms). After correction of susceptibility-induced [15] and eddy-current-induced geometric distortions and subject motion [14,16] using FMRIB Software Library (FSL) version 6.0 (www.fmrib.ox. ac.uk/fsl) [17], CS reconstruction recovered 257 unique DWIs from 112 undersampled DSI acquisitions [14,18]. FA and MD are estimated by voxel-wise model fitting using the Microstructure Diffusion Toolbox (MDT) [14]. A whole brain white matter mask was obtained from the T1-weighted MR image using the standard Freesurfer processing pipeline and refined through FA skeletonization [SMITH] [19] using the JHU-ICBM FA skeleton template [MORI] [20] provided with the FSL tool and thresholded at an FA value of 0.2. The mask was further corrected for white matter hyperintensities (WMH). WMH were automatically segmented with an in-house developed pipeline using DeepMedic [21,22], based on information from T1-weighted, T2-weighted, and FLAIR images.Applying this mask, global dMRI measures were computed as the average across voxels within normal appearing white matter (WM), i.e., white matter not classified as WMH. Additionally, WM tract-specific dMRI measures were derived for the regions of interest, that have been associated with cognition [23,24], provided by the JHU-ICBM DTI atlas [20].

**References**

1. Baddeley AD, Hitch G. Working Memory. *Current Biology*. Vol 20. 1974, 47–89.

2. Rey A. Mémorisation d’une série de 15 mots en 5 répetitions. In: Presses Universitaries de France (ed.). *L’examen Clinique En Psychologie*. Paris, 1970, 141–193.

3. Corsi PM. Memory and the Medial Temporal Region of the Brain. McGill University, 1972.

4. Mueller ST, Piper BJ. The Psychology Experiment Building Language (PEBL) and PEBL Test Battery. *J Neurosci Methods* 2014;**222**:250–9.

5. Coors A, Merten N, Ward DD *et al.* Strong age but weak sex effects in eye movement performance in the general adult population: Evidence from the Rhineland Study. *Vision Res* 2021;**178**:124–33.

6. Fischl B, Salat DH, Busa E *et al.* Whole Brain Segmentation. *Neuron* 2002;**33**:341–55.

7. Fischl B. FreeSurfer. *Neuroimage* 2012;**62**:774–81.

8. Buckner RL, Head D, Parker J *et al.* A unified approach for morphometric and functional data analysis in young, old, and demented adults using automated atlas-based head size normalization: reliability and validation against manual measurement of total intracranial volume. *Neuroimage* 2004;**23**:724–38.

9. Setsompop K, Gagoski BA, Polimeni JR *et al.* Blipped-Controlled Aliasing in Parallel Imaging (blipped-CAIPI) for simultaneous multi-slice EPI with reduced g-factor penalty. *Magn Reson Med* 2012;**67**:1210–24.

10. Cauley SF, Polimeni JR, Bhat H *et al.* Interslice leakage artifact reduction technique for simultaneous multislice acquisitions. *Magn Reson Med* 2014;**72**:93–102.

11. Xu J, Moeller S, Auerbach EJ *et al.* Evaluation of slice accelerations using multiband echo planar imaging at 3T. *Neuroimage* 2013;**83**:991–1001.

12. Menzel MI, Tan ET, Khare K *et al.* Accelerated diffusion spectrum imaging in the human brain using compressed sensing. *Magn Reson Med* 2011;**66**:1226–33.

13. Wedeen VJ, Hagmann P, Tseng WI *et al.* Mapping complex tissue architecture with diffusion spectrum magnetic resonance imaging. *Magn Reson Med* 2005;**54**:1377–86.

14. Harms RL, Fritz FJ, Tobisch A *et al.* Robust and fast nonlinear optimization of diffusion MRI microstructure models. *Neuroimage* 2017;**155**:82–96.

15. Andersson JLR, Skare S, Ashburner J. How to correct susceptibility distortions in spin-echo echo-planar images: application to diffusion tensor imaging. *Neuroimage* 2003;**20**:870–88.

16. Andersson JLR, Sotiropoulos SN. An integrated approach to correction for off-resonance effects and subject movement in diffusion MR imaging. *Neuroimage* 2016;**125**:1063–78.

17. Jenkinson M, Beckmann CF, Behrens TEJ *et al.* FSL. *Neuroimage* 2012;**62**:782–90.

18. Tobisch A, Schultz T, Stirnberg R *et al.* Comparison of basis functions and q‐space sampling schemes for robust compressed sensing reconstruction accelerating diffusion spectrum imaging. *NMR Biomed* 2019;**32**:1–2.

19. Smith SM, Jenkinson M, Johansen-Berg H *et al.* Tract-based spatial statistics: voxelwise analysis of multi-subject diffusion data. *Neuroimage* 2006;**31**:1487–505.

20. Mori S, Oishi K, Jiang H *et al.* Stereotaxic white matter atlas based on diffusion tensor imaging in an ICBM template. *Neuroimage* 2008;**40**:570–82.

21. Lohner V, Pehlivan G, Sanroma G *et al.* Relation Between Sex, Menopause, and White Matter Hyperintensities. *Neurology* 2022;**99**:E935–43.

22. Kamnitsas K, Ledig C, Newcombe VFJ *et al.* Efficient multi-scale 3D CNN with fully connected CRF for accurate brain lesion segmentation. *Med Image Anal* 2017;**36**:61–78.

23. Cremers LGM, de Groot M, Hofman A *et al.* Altered tract-specific white matter microstructure is related to poorer cognitive performance: The Rotterdam Study. *Neurobiol Aging* 2016;**39**:108–17.

24. Mielke MM, Okonkwo OC, Oishi K *et al.* Fornix integrity and hippocampal volume predict memory decline and progression to Alzheimer’s disease. *Alzheimer’s Dement* 2012;**8**:105–13.
